# Development of an MRI-compatible digital SiPM detector stack for simultaneous PET/MRI

**DOI:** 10.1088/2057-1976/2/1/015010

**Published:** 2016-02-04

**Authors:** Peter M Düppenbecker, Bjoern Weissler, Pierre Gebhardt, David Schug, Jakob Wehner, Paul K Marsden, Volkmar Schulz

**Affiliations:** 1 Imaging Sciences & Biomedical Engineering, King’s College London, UK; 2 Department of Physics of Molecular Imaging Systems, Institute of Experimental Molecular Imaging, RWTH Aachen University, Aachen, DE; 3 Philips Innovative Technologies, Aachen, DE; 4 Clinical Application Research, Philips Research, Aachen, DE; peter.dueppenbecker@kcl.ac.uk; volkmar.schulz@pmi.rwth-aachen.de

**Keywords:** PET/MRI, digital SiPM, MRI compatibility, time-of-flight, electromagnetic interference

## Abstract

Advances in solid-state photon detectors paved the way to combine positron emission tomography (PET) and magnetic resonance imaging (MRI) into highly integrated, truly simultaneous, hybrid imaging systems. Based on the most recent digital SiPM technology, we developed an MRI-compatible PET detector stack, intended as a building block for next generation simultaneous PET/MRI systems. Our detector stack comprises an array of 8 × 8 digital SiPM channels with 4 mm pitch using Philips Digital Photon Counting DPC 3200-22 devices, an FPGA for data acquisition, a supply voltage control system and a cooling infrastructure. This is the first detector design that allows the operation of digital SiPMs simultaneously inside an MRI system. We tested and optimized the MRI-compatibility of our detector stack on a laboratory test bench as well as in combination with a Philips Achieva 3 T MRI system. Our design clearly reduces distortions of the static magnetic field compared to a conventional design. The MRI static magnetic field causes weak and directional drift effects on voltage regulators, but has no direct impact on detector performance. MRI gradient switching initially degraded energy and timing resolution. Both distortions could be ascribed to voltage variations induced on the bias and the FPGA core voltage supply respectively. Based on these findings, we improved our detector design and our final design shows virtually no energy or timing degradations, even during heavy and continuous MRI gradient switching. In particular, we found no evidence that the performance of the DPC 3200-22 digital SiPM itself is degraded by the MRI system.

## 1. Introduction

Early investigations to combine positron emission tomography (PET) and magnetic resonance imaging (MRI) into truly simultaneous, hybrid imaging systems date back to the 1990s. Conventional PET detectors are inoperable inside an MRI, because they are based on photomultiplier tubes and are thus very sensitive to magnetic fields. First investigators circumvented this hurdle by inserting long optical fibres between the scintillators and the photomultiplier tubes. Thereby, the photomultiplier tubes could be operated in safe distance outside the main magnetic field (Garlick *et al* 1997, Shao *et al* 1997, Slates *et al* 1999). However, this approach limited itself to small prototypes, not only because the optical fibres degraded energy and time resolution, but rather because of the sheer amount of required fibres. More scalable and integrated approaches presupposed the replacement of the photomultiplier tubes by MRI-compatible detector types.

The next technological step was the use of avalanche photo diodes (APD). APDs are semiconductor devices and operate well inside strong magnetic fields (Pichler *et al* 1997). Numerous detector concepts were presented and multiple systems were successfully built up with this technology (Grazioso *et al* 2006, Schlyer *et al* 2007, Maramraju *et al* 2011). In 2010, the first commercial clinical whole-body simultaneous PET/MRI system was presented by Siemens Healthcare, based on APD technology. A disadvantage of APDs, however, remains their low intrinsic gain of typically only 10 to 1000 and their high temperature dependency of about ∼ 3.5% K^−1^ (Spanoudaki *et al* 2005). The resulting charge signal is in the order of only 10^−15^ C and requires a high gain and low noise charge amplifier, which makes APD-based detectors intrinsically prone to electromagnetic interference from the MRI system and special attention must be paid to electromagnetic shielding (Pichler *et al* 2006). The timing resolution of current APD detectors is in the range of several nanoseconds and thus insufficient for time-of-flight PET. Furthermore, APDs commonly require a bias voltage above 100 V and so require appropriate handling of high voltages.

A more advanced development of solid state photon detectors are arrays of Geiger-mode APDs. Those devices are known as silicon photomultipliers (SiPM) or multi pixel photon counters (MPPC). They provide a high internal amplification of 10^5^ to 10^6^ and fast response times. Coincidence timing resolutions well below 1 ns FWHM make them suitable for time-of-flight PET. In 2011, the first PET/MRI prototype systems based on silicon photomultipliers were presented: the Hyperion I system developed by Philips Research on behalf of the HYPERImage consortium (Schulz *et al* 2012, Weissler *et al* 2014) and a system developed at Seoul National University (Yoon *et al* 2012). Both scanner are prototype systems intended for small animal imaging. The first commercial clinical whole-body simultaneous PET/MRI system based on silicon photomultipliers was announced by GE Healthcare in 2014. Although silicon photomultipliers have been demonstrated to work inside MRI environments, they still require external signal digitization, e.g. by an application-specific integrated circuit (ASIC), and the capabilities of the digitizer used affect achievable system performance and integration substantially.

In 2009, a novel type of solid-state photon detector was presented, the so-called digital SiPM (Degenhard *et al* 2009, Frach *et al* 2009). The digital SiPM, also known as digital photon counter (DPC), enhances the idea of the common analogue SiPM by combining an array of Geiger-mode photodiodes with digital logic on a common silicon substrate. Its key feature is the detection and digitization of each individual photodiode breakdown. Thereby the previously analogue process of energy integration becomes a digital counting process. Furthermore, the digital SiPM contains a digital time stamper and thus requires no external digitization at all—recorded energy and time information is transmitted directly digitally for each detected event. The intrinsic coincidence time resolution of the digital SiPM is below 60 ps FWHM (Degenhard *et al* 2010) and therefore perfectly suited for time-of-flight PET.

Like the analogue SiPM, the operation of the digital SiPM is expected to be unaffected by strong magnetic fields and its intrinsic digitization should make it less prone to electromagnetic interference. The combination of its high performance, functional integration and expected MRI compatibility makes the digital SiPM a very promising device for building simultaneous PET/MRI systems. Based on Philips Digital Photon Counting DPC 3200-22 digital SiPMs, we developed an MRI-compatible detector stack and used it to build up small-animal simultaneous PET/MRI systems. This paper focuses on the development of the detector stack itself, first introducing basic MRI compatibility considerations and our detector design, followed by an in-depth characterization and optimization of its MRI compatibility.

## 2. MRI compatibility considerations

### 2.1. Interference with the MRI static magnetic field

The static main magnetic field *B*_0_, used to align nuclear spins, is the basic component of every MRI system. It is about 100 000 times stronger than the earth’s magnetic field and being present to all components inside an MRI bore it is the most obvious cause of interference effects. According to the Lorentz force law, a magnetic field deviates the path of charged particles in motion. In photomultiplier tubes this disturbs the process of electron multiplication and this was the primary motivation to replace photomultiplier tubes by solid-state detectors for simultaneous PET/MRI. Path lengths in solid-state detectors are much shorter, so that they can remain functional even within strong magnetic fields, although they still can be affected, e.g. by magnetoresistance. Numerous electronic components, like resistors, capacitors and air coils, are empirically known to operate well within magnetic fields, however others, e.g. ferrite coils, lose most of their functionality, because of ferromagnetic saturation.

The other aspect of the main magnetic field is its influence on MRI image quality. Besides the field strength, the field homogeneity within the field of view is crucial for MRI image quality, because any field inhomogeneity shifts the resonance frequency locally and results in signal loss and image artefacts. This becomes especially obvious for gradient echo based imaging sequences, as phase recovery gets distorted and distortions accumulate for multiple echoes (Brown and Semelka 2010). Consequently, common MRI systems require a very homogeneous main magnetic field that deviates not more than a few parts per million within the field of view. Field inhomogeneities can be caused by any change in magnetic susceptibility within the main magnetic field, but ferromagnetic materials, which obtain a very high and non-linear magnetic susceptibility, are especially problematic (Schenck 1996). The common primary design strategy is to minimize field inhomogeneities by proper material selection, in particular by eliminating ferromagnetic materials.

A further source of field inhomogeneities are stray magnetic fields caused by electrical currents. Even though their existence is unavoidable, the influence of stray magnetic fields can be minimized by placing supply and return currents close together, so that resulting fields cancel out, e.g. by using a strict star topology, careful circuit board routing, and the use of twisted pair or coaxial cables.

### 2.2. Interference with the MRI gradient system

MRI spatial encoding is based on shifting the resonance frequency spatially and temporally by superimposing magnetic gradient fields to the static main magnetic field. Although the absolute field strength of MRI gradients is about two orders of magnitude lower than the static magnetic field, they can cause major interference effects, because the gradients are switched very rapidly, up to several hundred times per second. According to Faraday’s law of induction, a change in magnetic flux causes a reverse induced current in closed electrical paths, so-called eddy currents. Eddy currents can influence electronic components, cause heating and vibrations. Furthermore, they generate reverse magnetic fields that distort the intended gradient fields and thus cause image artefacts. To minimize eddy currents and resultant distortions, conductive structures should be minimized and especially large conductive loops and planes should be avoided, either by geometry or selection of materials.

Common MRI systems contain gradient systems for *x*, *y*, and *z* encoding. Naming and specification of MRI gradients refers solely to the axial component of the resulting magnetic field. However, because of the solenoidal property of magnetic fields, the MRI gradient system generates additional radial field components. Those radial components are usually negligible for MRI imaging, but they have to be considered for MRI compatibility. Typically, PET electronics are located tangential to the field of view and are thus perpendicularly aligned to radial field components. This is especially true for the *z*-gradient along the axial direction, which is commonly generated by an antiparallel pair of Helmholtz coils. This coil configuration generates additional radial magnetic components with comparable magnitude alike the intended axial component.

Within the field of view, the magnetic fields generated by MRI gradients increase with distance to the MRI isocentre. Consequently, the change in magnetic flux density over time and resulting gradient interference effects depend on position and become stronger with increasing distance.

### 2.3. Radio frequency (RF) interference between PET and MRI system

RF interference between the PET and the MRI system can occur in both directions. On the one hand, RF excitation pulses generated by the MRI might couple into the electronics of the PET detector and disturb or damage it. On the other hand, the PET detector will emit RF radiation during operation, which could couple into the receive chain of the MRI. Electromagnetic shielding of the PET detector is a possible solution. However, common RF shielding requires high conductivity materials, e.g. copper foil, which are in turn problematic in that they can result in gradient interference. Thus, the primary goal is to minimize RF emission by following good engineering practice and avoid the propagation of high frequency voltage and current variations as well as antenna-like structures. At the same time this reduces the risk of coupling RF power from the MRI into the PET detector.

The relevant frequency range of the MRI is determined by the field strength and the Larmor frequency of the nucleus being imaged. The centre frequency for protons at 3 T is 127.28 MHz. This frequency is of the same order as the operating frequencies of many PET detector components, e.g. the clock of the digital SiPM is by default supplied at 200 MHz and most digital processing is done at 100 MHz. Furthermore, harmonics from lower frequency switching, e.g. caused by a switch mode power supply (Wehner *et al* 2014), can also interfere with the MRI system. Thus also the selection of operating frequencies influences RF interference.

## 3. Design of the digital SiPM detector stack

**Figure 1.**
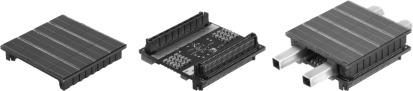
MRI-compatible digital SiPM sensor tile, interface board and assembled detector stack with cooling pipes. The edge length of the quadratic sensor tile is 32.7 mm.

Our detector stack is composed of three assembly groups: the sensor tile, the interface board and two cooling pipes (figure 1). The sensor tile contains the digital SiPMs, whereas the interface board acts as a data acquisition and control unit with an interface to the singles processing unit presented by Weissler *et al* (2012b).

The top side of the sensor tile contains an array of 8 × 8 digital SiPM channels with 4 mm pitch using Philips Digital Photon Counting DPC 3200-22 devices. The DPC 3200-22 combines four digital SiPM channels on one silicon die, the four channels sharing a common trigger, time stamping and communication unit. Each silicon die is mounted with a conductive glue onto the sensor tile and up to 48 bond wires along the top and bottom edge of each die connect the power and signal lines. Each DPC 3200-22 die is connected to a dedicated clock and a synchronization line, which allows the configuration of a fine grained trigger and clocking network. On the bottom side the sensor tile is equipped with decoupling capacitors, a digital temperature sensor, a 16 Mbit flash memory, and two 80-pin connectors. Both connectors connect the sensor tile to the interface board with a stacking height of 6 mm. The sensor tile is split into two galvanically isolated halves to avoid a conductive loop through the connectors and the interface board. Although similar in functionality and dimensions compared to a conventional digital SiPM tile as presented by Degenhard *et al* (2010), our sensor tile is an entirely new design, optimized for MRI compatibility and reliable cooling.

The central component of the interface board is a Xilinx Spartan 6 XC6SLX45 FPGA. It collects all measurement data, distributes clock and synchronization signals, configures the digital SiPMs and controls the supply voltage regulators. The interface board contains a digitally adjustable voltage regulator for the bias and reset voltage and a fixed 1.8 V regulator for the supply of the detector stack. In course of optimization, we later on added a fixed 1.2 V regulator for the FPGA core voltage to stabilize the supply voltage during gradient switching. The bias and reset voltage can be adjusted in 256 steps from 15 V to 34 V and 3 V to 4 V, respectively. Additionally, both supplies can be switched on/off and include a voltage and current monitor, as well as a hard-wired current limiter to protect the digital SiPMs in case of malfunction. The remaining space on the interface board is used for decoupling capacitors.

**Figure 2.**
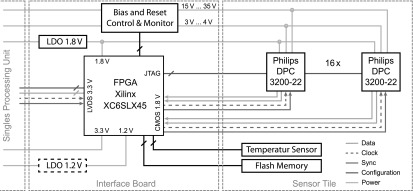
Functional overview of the detector stack. The sensor tile contains the digital SiPMs, a digital temperature sensor and a Flash memory. Data acquisition, configuration and power supply is controlled by the interface board. Acquired measurement and control data is bundled into one communication stream and forwarded to the singles processing unit. The low-drop voltage regulator (LDO) for the 1.2 V supply was added during optimization of the detector stack.

A functional overview of the whole detector stack is given in figure 2. Further implementation details of the sensor tile and the interface board are presented in figure 1 in the supplement. The data readout platform is described in Gebhardt *et al* (2012).

The detector stack is intended to operate in enclosed module boxes. This mandates a reliable cooling system, if only for the reason to prevent overheating. Beyond that, the performance of the digital SiPM depends on temperature. Although the temperature dependency of the digital SiPM is in principle lower than that of analogue SiPMs (Frach *et al* 2009), temperature directly affects the breakdown voltage, the dark count rate and the switching characteristics, and thus influences the photon detection efficiency, the dead time and the velocity of the integrated time stamper of the digital SiPM. For this reason, our system design included a liquid cooling system from the very beginning.

The basic elements of the cooling system are two rectangular brass pipes between the interface board and the sensor tile. Thermal pads couple those brass pipes to dedicated cooling surfaces on the interface board and the sensor tile. The cooling pipes are connected to a liquid cooling system with a monoethylene glycol and water mixture using non-spill and non-magnetic connectors. More than 600 vias through the circuit board of the sensor tile improve thermal coupling between the digital SiPMs and the cooling pipes. All voltage regulators are placed directly opposed to the cooling pipes on the bottom side and are connected via thermal vias to the cooling surfaces.

A major concern was to minimize *B*_0_ distortions by avoiding ferromagnetic components. However, the majority of electronic components contain nickel in their surface finishes or iron in their lead frames. The availability of dedicated non-magnetic components is very limited and custom-made, non-magnetic components are in many cases impracticable because of high development costs, especially for small lot sizes. We thus strived to use selected non- to weakly magnetic standard components wherever possible. The selection process was primarily based on screening components with permanent magnets and acquiring *B*_0_ field maps by MRI measurements, because information about magnetic properties, e.g. from data sheets, is very limited. Only the connectors and most capacitors are explicitly non-magnetic components.

Capacitors play a twofold role in MRI-compatible electronic design: They are required to provide a stable power distribution network and thus can influence RF interference, but they are also a major source of *B*_0_ distortions, because the electrodes of today’s commonly used multi layer chip capacitors are usually made of nickel. Only few vendors offer non-magnetic capacitors and available case sizes, or rather capacitance density, is severely limited compared to standard components. Large case sizes are undesirable, not merely because of construction space, but rather because of increased lead inductance, which dominates net component impedance at higher frequencies. Wherever possible, we used non-magnetic capacitors in the smallest available package, however, the interface board still contains some high-capacity magnetic capacitors in the smallest available case size, because suitable non-magnetic capacitors were unavailable.

Another source of ferromagnetic material is nickel contained in the surfaces finish of the circuit boards. Both circuit boards are fabricated with a nickel immersion gold (ENIG) finish, which is an established and widely used surface finish in electronic industry and provides a very reliable surface for wire bonding. Having a reliable surface for wire bonding is crucial, because all signal and power lines of the digital SiPM are wire-bonded and a single bond fault can cause malfunction of an entire sensor tile. Although selected suppliers offer bond-able nickel free surface finishes, these were not verified for wire-bonding of the digital SiPM at design time.

The circuit boards are designed with particular care to reduce electromagnetic interference. Return paths for signal and power lines are placed close together, including the pin assignment of the connectors. Where possible, signal lines are routed on inner layers. Low impedance connection of decoupling capacitors and power pins to the supply planes took highest priority during all design phases.

## 4. Experimental setup

For testing, the detector stack is mounted on a singles processing unit (Weissler *et al* 2012b), which supplies power and provides the communication interface to exchange data via an optical gigabit Ethernet link with a data acquisition and control computer. Furthermore, the singles processing unit distributes the reference clock signal from an optically-connected external clock source. Raw detector and status data, including data from the temperature sensor and voltage monitors, is stored on disk and post-processed with Java and Matlab. To reduce the dark count rate and dead time, 20% of the most noisy digital SiPM cells were disabled. All measurements were carried out with 2.5 V overvoltage and the cooling temperature was set to approximately 10 °C.

**Figure 3.**
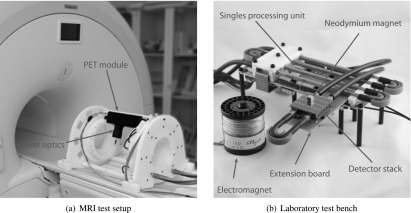
(a) For testing purposes the detector stack is mounted in a PET module on a gantry. The black build-up contains the laser optics and is connected via an optical fibre to the laser unit outside the MRI room. (b) Test bench with detector stack, extension board and singles processing unit. Shown magnets were used to simulate static and dynamic magnetic fields, without requiring an MRI system. The extension is a straight-forward connection of all connector pins between the interface board and the singles processing unit to simplify the installation of test equipment. It was only used for experiments outside the MRI.

Measurements inside the MRI were performed on a 3 T Philips Achieva MRI scanner with 60 cm bore diameter. The detector stack and the singles processing unit are therefore mounted in a PET module on a gantry, as shown in figure 3(a), resulting in the detector stack being positioned approximately 11 cm above the isocentre of the MRI. Measurements outside the MRI were performed with a detector stack mounted on a bare singles processing unit in a light-tight cabinet and, to simplify the installation of test equipment, the connector between the detector stack and the singles processing unit was extended with an extension board, as shown in figure 3(b). The extension board is a straightforward extension of all connector pins between the singles processing unit and the detector stack and does not contain any active components.

To be independent of a specific scintillator configuration, we illuminated the detector stack with short laser pulses instead of scintillation light. The use of laser pulses eliminates the randomness of radioactive decay from our experiments and a second detector as timing reference becomes redundant. Furthermore, the use of laser pulses with a fixed frequency constitutes a uniform sampling in time. This not only allows a direct time domain analysis down to single events, but also offers the opportunity for frequency domain analysis using Fourier transformation, which is not possible with random events generated by radioactive decay.

**Figure 4.**
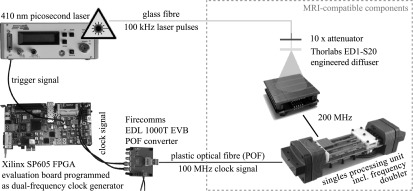
Clocking infrastructure used for synchronized laser excitation of the detector stack. The FPGA evaluation board is used as low-jitter, dual-frequency clock generator to generate the reference clock and the trigger signal. Optical fibres allow the operation of the laser and clock generator safely outside the MRI room.

As illustrated in figure 4, laser pulses are generated by an advanced laser diode systems EIG1000D 410 nm pulsed picosecond laser and are coupled into a 25 m long optical fibre. At the opposite end of the fibre, laser pulses are coupled out and attenuated 10 times by a neutral density filter to achieve photon count values similar to scintillation light. A Thorlabs ED1-S20 microlens diffuser widens the laser pulses before they hit the detector. To illuminate the detector stack inside a closed PET module, we milled a 30 mm hole into the housing and attached a fixture to hold the optical fibre, the attenuator and the diffuser.

Each laser pulse is triggered by an electrical trigger signal. Measurements presented in this paper were performed with a trigger rate of 100 kHz to prevent saturation of the data readout chain, whereas the reference clock for the digital SiPMs is supplied at 100 MHz to the singles processing unit and is later on doubled to 200 MHz inside the FPGA of the interface board. Consequently, any jitter or drift between the trigger signal and the digital SiPM reference clock will directly influence measured timestamps. Because a suitable low-jitter, dual-frequency clock generator was not available, we programmed a Xilinx SP605 FPGA evaluation board to generate the trigger signal and the reference clock. Both signals are derived with a counter from the same on-board oscillator and run synchronously with a fixed phase relationship. Measured jitter between the reference clock and the trigger signal was 22 ps FWHM (figure 2 in the supplement). The trigger signal for the laser is directly connected to the trigger input of the laser unit, whereas the reference clock signal is connected to a Firecomms EDL 1000 T-EVB plastic optical fibre (POF) converter and transmitted via a POF to the singles processing unit.

## 5. Methods used to measure PET/MRI interference

### 5.1. Quantification of *B*_0_ distortions

Quantification of *B*_0_ distortions followed the phase imaging technique as described in the ACR MRI Quality Control Manual (Weinreb *et al* 2004). Test objects were placed centred on a cylindrical, oil-filled image quality phantom with an inner height of 10 cm and a diameter of 38 cm (see figure 3 in the supplement). We tested the detector stack and its individual components as well as a scintillator array, whereby the alignment of test objects was consistent to their intended mounting position on a PET ring. Presented images are transverse 5 mm thick slices centred through the device under test with a pixel size of 1.76 mm × 1.76 mm, representing the *B*_0_ shift caused by the respective test object. Our investigations are independent of a specific field of view and focus on the spatial extent of distortions of single components. Thus, presented figures show and compare plain field maps, but are corrected for distortions caused by the phantom itself by subtraction of a reference scan of the phantom without any test object placed on it.

### 5.2. *B*_0_ influence on detector stack

To investigate the influence of static magnetic fields, we operated the detector stack inside a 3 T Philips Achieva MRI system. However, the large extend of the MRI static magnetic field impedes selective exposure of individual components of a device in operation and testing of individual components in operation within an MRI is limited by component interdependencies. Thus, to identify components that are sensitive to static magnetic fields, we additionally used tiny neodymium magnets with edge lengths of 4 mm to 10 mm. The measured field strengths of the magnets used were about 0.5 T at the poles and 0.25 T along the sides. This is clearly lower than in a 3 T MRI system and consequently achievable effects will also be weaker. However, this is secondary for our purpose, because the aim of using permanent magnets is the identification of critical components rather than quantification of interference effects.

### 5.3. Gradient influence on detector stack

In previous experiments, the *z*-gradient caused the strongest interference effects and therefore our investigations focused on the influence of the *z*-gradient (Wehner *et al* 2014). An echo planar imaging (EPI) sequence with artificially high *z*-gradient switching served as a worst-case scenario to emphasize interference effects. Obtained *z*-gradient slew rate of the test sequence is 198 T m^−1^ s^−1^ at a maximum gradient strength of 30 mT m^−1^. Echo and repetition time are set to TE = 12 ms and TR = 24 ms, the shortest possible values. The EPI factor is set to 49, which results in an almost continuously switching of the readout gradient during the whole sequence. This sequence doesn’t produce any valuable MRI images. It is solely intended to create a worst-case scenario to investigate the influence of gradient switching on the detector stack.

Inside an MRI system, the installation of measurement equipment and the selective exposure of individual components is difficult. Similar to the use of permanent magnets, we were looking for a simple solution to simulate locally limited magnetic gradient fields without requiring an MRI system. Therefore we built up an electromagnet with 190 turns of copper strand on a 20 mm core, driven by an audio power amplifier and a signal generator. The coil carrier contains an axial hole with 10 mm diameter, which allows the illumination of a small area of the detector stack through the coil. This configuration produces slew rate amplitudes up to 260 T s^−1^ using a 10 kHz, 2.5 A rms, sinusoidal waveform. In close proximity to the core of the coil, the change in magnetic flux is thus even higher than the MRI system used can provide, but the spatial extend is very limited, as intended.

### 5.4. RF interference

RF interference measurements were performed using the spurious signal sequences provided by the service tools of Philips Achieva MRI systems. This sequence is a modified spin echo imaging sequence with disabled gradients and RF power reduced to a minimum. Essentially, the MRI system is used as a receiver only. Image data was exported as DICOM data and post processed with MATLAB to extract the noise distribution as a function of frequency.

The RF investigations presented in this paper are intended to demonstrate the significance of a proper decoupled power distribution network for RF interference with the MRI system. We therefore removed all decoupling capacitors of a sensor tile and compared the resulting RF noise figures to a fully equipped sensor tile. To emphasis the effect, the RF shielding of the detector module was replaced by an RF-transparent glass fibre housing. The MRI coil used was a local transmit and receive, 12-leg-birdcage resonator, identical as described in Wehner *et al* (2015).

## 6. Results

### 6.1. *B*_0_ distortions caused by detector stack

**Figure 5.**
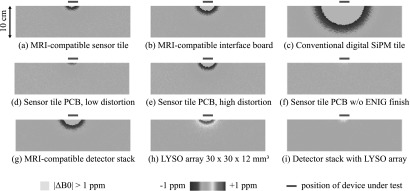
*B*_0_ distortion maps. Shown are transverse slices. For proper visualization and ease of comparison, the colour coding range is limited to ±1 ppm and distortions stronger than 1 ppm are greyed.

Figure 5 summarizes the results of the *B*_0_ distortion quantification. As shown in figures 5(a)–(c), the MRI-compatible sensor tile and interface board cause considerably less *B*_0_ distortion than a conventional digital SiPM tile, that was not optimized for MRI compatibility. A subsequent detailed analysis obtained by disassembling multiple sensor tiles and rescanning individual components revealed that the remaining distortions are dominated by the bare circuit boards, though the amount of distortion varies for different batches, as apparent from figures 5(d) and (e). By design, the ENIG surface finish used contains a nickel layer and was thus suspected of causing the distortions. figure 5(f) confirms this assumption: after grinding the ENIG layer off, the remaining circuit board with bare copper traces caused no ferromagnetic distortions any more.

Figure 5(g) shows the *B*_0_ distortions caused by an assembled detector stack, composed of an interface board and a sensor tile. The distortions of the detector stack are clearly dominated by ferromagnetic materials. In contrast, common scintillator materials for PET—in particular BGO and LSO—are diamagnetic (Yamamoto *et al* 2003). Verification of LYSO by scanning a pixelated 30 × 30 × 12 mm^3^ LYSO array, as shown in figure 5(h), confirmed that LYSO behaves diamagnetically as well and causes distortions with the opposite sign. In their intended application, the detector stacks will be placed on top of the scintillator array and thus both effects will superimpose. Figure 5(i) shows the net *B*_0_ distortions caused by the detector stack in combination with the LYSO array. In this particular configuration both distortions almost cancel out.

### 6.2. *B*_0_ influence on detector stack

**Figure 6.**
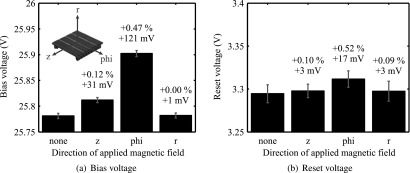
Directional effects of a 3 T magnetic field on the voltage regulators of the bias (a) and reset voltage (b). Whereas magnetic fields perpendicular to the circuit boards (*r*) cause almost no effect, magnetic fields parallel to the circuit boards (*z*, phi) do. The given directions relate to the cylindrical coordinate system of the MRI and the PET module installed in default position. Actual measurement data were obtained by rotating the PET module inside the MRI bore. Red bars indicate the standard deviation of the measured voltage noise floor.

Operating the detector stack inside the main magnetic field of the MRI revealed slightly decreased photon count values, as reported in Düppenbecker *et al* (2012). This effect could be traced back to a shift of the bias voltage, which directly influences the photon detection efficiency of the digital SiPM. As shown in figure 6(a), the effect is directional. Depending on the alignment, the originally set bias voltage of 25.78 V shifted by up to 121 mV (0.47%), although the shift in the default installation position is comparable low with 31 mV (0.12%). In particular, magnetic fields parallel to the circuit board cause a voltage shift, whereas perpendicular magnetic fields show no significant effect. The same voltage regulator is used to control the reset voltage and measurements presented in figure 6(b) confirm a similar behaviour. Although the observed absolute shift is about eight times smaller, the relative change is comparable and suggests a gain dependency.

Probing individual components with small permanent magnets partially reproduced the effects measured above and identified the low drop voltage regulators as being sensitive to magnetic fields. Measurements with different gain settings and magnet orientations (figure 4 in the supplement), confirmed that the observed voltage shift scales with the set gain of the voltage regulator and depends on the applied field direction.

### 6.3. Gradient influence on detector stack

#### 6.3.1. Gradient influence on bias voltage and energy resolution

**Figure 7.**
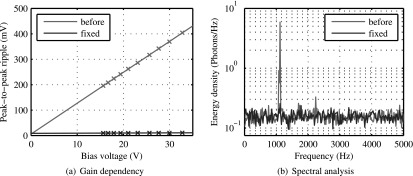
(a) Ripple induced with the electromagnet on the bias voltage measured before and after stabilizing the voltage regulator. The gain dependency of the ripple indicates, that induced voltage variations get amplified by the voltage regulator. (b) Spectral analysis of photon count values over time during MRI gradient test sequence. The main peak at 1120 Hz corresponds to the main gradient switching frequency of the used MRI test sequence. After stabilizing, the main peak is reduced by more than 27 dB.

As we previously reported (Wehner *et al* 2015), the energy resolution of PET measurements decreased during MRI gradient switching. This effect correlates with an instability of the bias voltage during gradient switching and is explained by the direct impact of the bias voltage on photon detection efficiency. The same effect is reproducible in lab by means of simulating MRI gradients with an electromagnet. Repeating the measurement at different bias voltage levels, as presented in figure 7(a), revealed a linear relationship between the set bias voltage and the voltage ripple. This indicates, that the ripple is injected into the control loop of the voltage regulator and thus gets amplified, depending on the set bias voltage.

Based on this finding, we redesigned the control loop of the regulator and stabilized it with an additional feedforward capacitor. This reduced the voltage ripple significantly and stabilized energy resolution. Measured energy resolution with laser excitation using the unmodified interface board was 10.6% without gradient switching and degraded to 11.0% during application of the MRI gradient sequence (see figure 5 in the supplement). The improved interface board showed 10.5% energy resolution in both cases. A spectral analysis of photon counts over time during MRI gradient switching points out the effect more clearly, as shown in figure 7(b). The unmodified interface board shows a main peak at 1120 Hz, which corresponds to the main switching frequency of the readout gradient. After stabilizing, the main peak is reduced by more than 27 dB and close to the noise level.

Although the reset voltage regulator is based on the same topology as the bias voltage regulator, the amplification factor and thus the resulting voltage ripple is about eight times lower and we couldn’t attribute any consequences to that. Nevertheless, for reasons of precaution we redesigned the reset voltage controller in the same way.

#### 6.3.2. Gradient influence on timing

**Figure 8.**
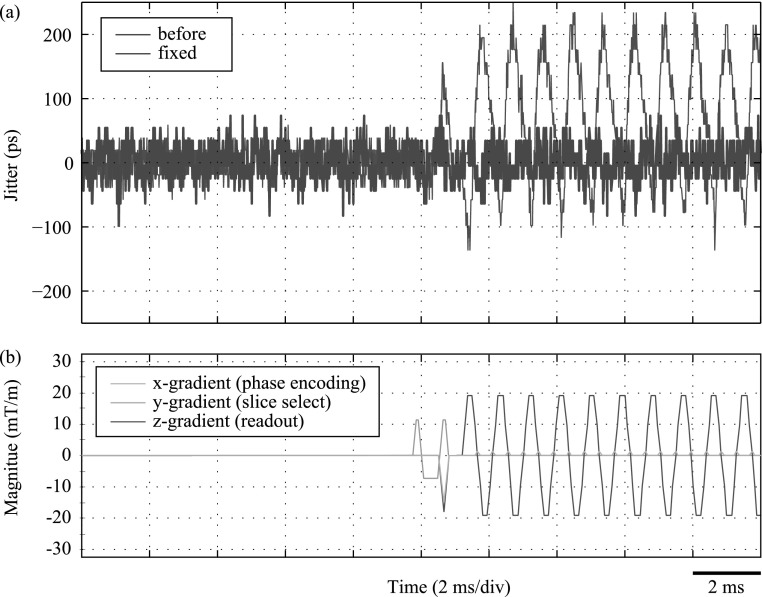
Measured timing jitter at start of gradient test sequence (a) and corresponding gradient sequence diagram (b). The initial design was affected by timing jitter up to 250 ps and the time course of jitter is directly correlated with the gradient sequence. In contrast, the improved interface board with local core voltage control operates stable during gradient switching.

The red curve of figure 8 shows the timing jitter of a single digital SiPM channel during the start of the gradient test sequence. It reveals that individual timestamps deviate up to 250 ps during MRI *z*-gradient switching and that the jitter directly correlates with the gradient pulses.

Simulated sinusoidal gradient fields with an electromagnet reproduced similar effects. Repeated measurements at different positions showed, that the magnitude of timing jitter varies with the position of the electromagnet. The timing jitter was strongest when the electromagnet was placed on the extension board, rather than directly on the detector stack. This indicated, that the primary source of timing jitter is not induced in the detector stack itself but in the supporting infrastructure, and subsequently propagates, e.g. via the power rails or the clock signal.

Figure 10 shows the output clock signal, measured with an oscilloscope at one of the connectors towards the sensor tile. It clearly depicts, that the output clock is affected by phase jitter during gradient switching, but the shape and the amplitude of the clock signal are preserved. However, an examination of the input clock signal revealed, that the clock signal enters the interface board cleanly (figure 6 in the supplement). Also a change of the slew rate settings of the clock output drivers did not show any effect (figure 7 in the supplement). Altogether, this suggests that the distortion of the clock signal happens inside the core logic of the FPGA. The FPGA output drivers for the clock signals are powered by the 1.8 V supply rail, whereas the internal logic of the FPGA is powered by the 1.2 V rail supplied by the singles processing unit. For testing purpose, we thus bypassed the 1.2 V supply and connected an external laboratory power supply directly to the interface board. This eliminated the previously observed timing jitter.

**Figure 9.**
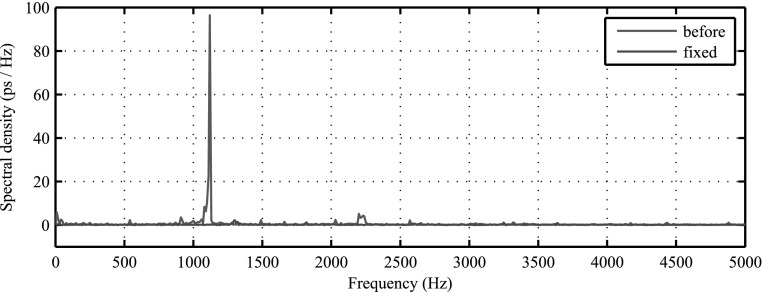
The spectral analysis of timing jitter clearly reveals the main gradient switching frequency of the MRI gradient test sequence. The fixed interface board with an additional voltage regulator for the core voltage supply of the FPGA is immune to this effect.

To verify this achievement inside the MRI, a dedicated voltage regulator for the 1.2 V supply had to be integrated onto the interface board, which required a slight redesign and a new production run of the circuit boards. Following measurements inside the MRI confirmed previous laboratory measurements: the timing resolution remains stable, even during heavy and continuous gradient switching, as proven by the blue curve in figure 8. A spectral analysis as shown in figure 9 reveals the effect even more clearly: whereas the jitter spectrum of the initial design (red curve) shows a clear peak corresponding to the main gradient switching frequency, the improved design (blue curve) shows an almost flat jitter spectrum.[Fig bpexaa05b0f10]

**Figure 10. bpexaa05b0f10:**
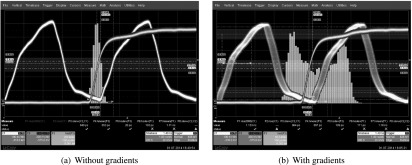
Clock jitter measured at the connector of the interface board without (a) and with gradients (b). The red curve shows the trigger signal of the picosecond laser and the yellow curve is the measured clock output signal at the connector of the interface board. The shape and magnitude of the clock signal is preserved during gradient switching, but the signal is shifted in phase. The blue histogram shows the jitter distribution. Gradients were applied with a small electromagnet. The time scale is 1 ns per division for the clock and trigger signal and 100 ps per division for the histogram data.

### 6.4. RF interference

**Figure 11.**
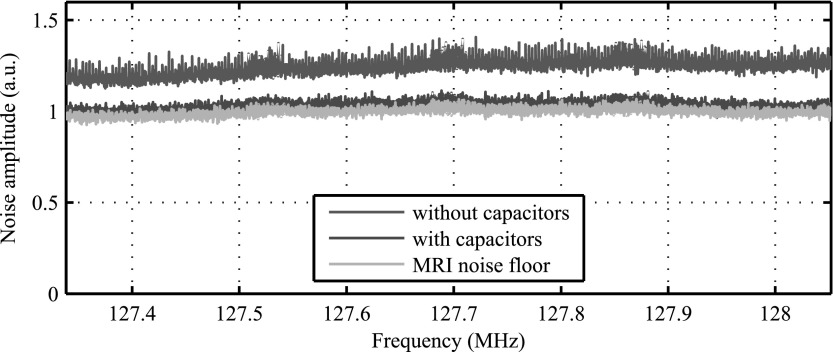
RF noise figure of a single detector stack with and without decoupling capacitors. To emphasise the effect, the detector stack was mounted on a PET module without RF shielding.

In figure 11, the noise floor of a regular sensor tile in operation is compared to a sensor tile with all decoupling capacitors removed. Without decoupling capacitors, the noise floor increased by about 25% and digital noise patterns became visible. The measurement was carried out with a single detector stack on a PET module without RF shielding to demonstrate the effect more clearly.

## 7. Discussion

*B*_0_ distortions caused by our MRI-compatible detector stack are significantly reduced compared to a conventional design. Apart from most capacitors and connectors, the improvements were mainly achieved by careful selection of non- to weakly-magnetic standard components. Especially for small batches, the use of standard components is beneficial to control costs and lead times. Remaining ferromagnetic distortions attributable to the sensor tile are dominated by the surface finish of the circuit boards and could be further reduced in future designs, either by using a nickel-free surface finish or by reducing the amount of gold-plated areas. However, the amount of nickel contained in the surface finish varies because of process variations and is difficult to predict.

The fact that nowadays common PET scintillators are diamagnetic can be used to compensate for distortions caused by ferromagnetic materials. In our example, the diamagnetic effect of LYSO compensates to a great extent for the distortions caused by the detector stack. The complete avoidance of ferromagnetic materials for an MRI-compatible PET detector with BGO, LSO or LYSO scintillators is therefore not essential and a certain amount of ferromagnetic components is actually desirable.

Easing the requirements on non-magnetic materials could be particularly beneficial for capacitor selection. Capacitors were shown to have a two-fold impact on MRI compatibility: they influence *B*_0_ distortions and RF interference. Selected suppliers offer non-magnetic capacitors, but available capacity densities are very limited and they are expensive. If carefully used, standard capacitors with nickel electrodes in the smallest available packages might be a viable choice to reduce construction space, costs and lead times. MRI compatibility thus clearly benefits from the trend to miniaturization of electronic components.

Nevertheless, one major issue remains: the unspecified magnetic properties of most standard components make it difficult to control *B*_0_ distortions in a production environment. The approach of tightly specifying magnetic properties of all components may sound obvious, but the required effort should not be underestimated. Adapted passive shimming by either additional diamagnetic, paramagnetic or ferromagnetic materials might be a possible solution to compensate for variations, although it introduces additional complexity. In the end, a combination of both approaches might provide the most viable and cost-effective solution.

Magnetic fields caused voltage regulators to drift, but the drift does not affect detector performance itself, because it can be easily compensated for by performing detector calibration inside the MRI, e.g. determination of breakdown voltage and operating point. However, the observed effects depend on detector orientation and are likely to increase at higher field strengths and should therefore be carefully considered for future developments. The results presented do not reveal the underlying physical effects to explain the observed deviations. To correctly interpret the directional dependency, it would require detailed information of the internal circuit layout, which was not available to us. According to the data sheets, both voltage regulators use an internal 0.6 V voltage reference. Considering the observed gain dependency, it suggests that the magnetic field directly impacts the voltage reference or the corresponding comparator circuit. Consequently, a very broad range of devices could be susceptible to magnetic fields, e.g. all devices using a voltage reference or comparators. All such devices might in principle remain operational inside an MRI, but their operating range and performance could be affected.

Gradient-induced voltage variations influenced the detector stack most seriously, although the induction itself took place in the supporting infrastructure and propagated via the power rails to the detector stack. In particular, the effect on the FPGA core logic is remarkable. Variations of the supply voltage affected the switching characteristics of the FPGA core logic and lead to phase jitter. We identified this effect, because we had special interest in timing resolution. However, changing switching characteristic will reduce the jitter margin of any digital logic and could thus cause various malfunctions. In practice, actual effects might be more concealed, e.g. increased bit error rates on data links.

Inexpensive magnets—tiny permanent magnets and a self-wound electromagnet—turned out to be efficient ways of investigating B_0_ and gradient interference. Both allow the induction of interference effects without requiring access to an MRI, which usually is a very expensive and scarce resource, and thus help to shorten development cycles. The field range of both magnets is small compared to an MRI and thereby allows selective testing and identification of critical components. Furthermore, working with small magnets on a test bench allows the use of test equipment, without worrying about MRI interference or safety issues.

The synchronized excitation with laser pulses proved to be very powerful. It allows the measurement of effects at single event level, enables frequency domain analysis and thus reveals effects that are otherwise hard to measure. In particular, frequency domain analysis can be very sensitive and useful to trace back characteristic frequency components.

The presented measurement setup is MRI-compatible, because only passive, optical components enter the MRI room. This is a huge advantage compared to electrical test equipment, e.g. an oscilloscope with active probes, which is subject to MRI interference itself. Beyond PET/MRI interference characterization, the synchronized laser excitation method could also be used to support the development of future time-of-flight PET systems: as time resolution strives towards 100 ps and below (Schaart *et al* 2010), clock jitter, caused by whatever source, becomes increasingly important.

Being able to measure the effect at a single event level opens up entirely new possibilities: we used this knowledge to optimize and eliminate initially observed degradations, but it could also be used to calculate the influence on single events caused by specific MRI sequences. This potentially offers the opportunity to compensate interference effects during post-processing, e.g. in case that interference effects are unsolvable or too costly to fix.

## 8. Conclusion

The developed detector stack allows the operation of digital SiPMs simultaneously and stably inside a 3 T MRI system. In our experience, the digital SiPM simplifies system integration and MRI compatibility, because it requires no external digitization and directly interfaces to an off-the-shelf FPGA. This reduces the risk to couple in electromagnetic distortions, saves—potentially magnetic—components as well as construction space. Furthermore, the low and positive bias voltage of about only 30 V allows the design of a very compact bias voltage supply with standard components and tight circuit board routing.

With the development of the detector stack we follow a very aggressive approach to integrate as much as possible detector infrastructure inside the MRI. This gives the advantage of less cabling and scales more easily with increasing system geometries and requirements. Although the MRI is a very challenging environment to operate electronic equipment, we showed that it is possible without sacrificing detector performance. The required detector electronics do not necessarily have to be more complex compared to conventional ones, but careful design and verification is required.

Observed *B*_0_ effects on voltage regulators are notable, but do not impact detector performance. Most severe interference effects were caused by gradient-induced supply voltage variations and initially caused a degradation of energy and timing resolution. We identified and eliminated the root cause of both effects and after optimization the detector stack operates very stably without performance degradations, even during heavy and continuous gradient switching. All observed MRI interference effects could be ascribed to the detector infrastructure. In particular, we found no evidence that the performance of the DPC 3200-22 digital SiPM itself is degraded by the MRI system.

## 9. Outlook

The detector stack was developed to facilitate good system integration and we already used it to build three preclinical PET/MRI systems (Weissler *et al* 2012a). The systems are in use to evaluate the performance and PET/MRI interference of the digital SiPM on system level (Schug *et al* 2015a, 2015b, 2015c, Wehner *et al* 2015), including preclinical imaging studies (Weissler *et al* 2015), and further research is ongoing.

The next development steps of the detector stack should focus on reducing the overall stack height, which is important for further integration into small animal as well as whole body clinical systems. This could, for example, be achieved by an integration of the interface board with the singles processing unit and could reduce the total height of the PET detector electronics to less than 10 mm. Although the detector stack was developed for PET/MRI, the presented design is also suitable for SPECT/MRI or PET only systems.

Future PET/MRI system designs might position the PET detector closer to the MRI gradient and RF systems, and eventually also to higher field strengths. It is likely that this will emphasise interference effects and may requires further optimization. The methods presented in this paper should provide the right tools to support those developments.
